# Shell resource partitioning as a mechanism of coexistence in two co-occurring terrestrial hermit crab species

**DOI:** 10.1186/s12898-019-0268-2

**Published:** 2020-01-16

**Authors:** Sebastian Steibl, Christian Laforsch

**Affiliations:** Department Animal Ecology I, University of Bayreuth and BayCEER, Universitaetsstr. 30, 95440 Bayreuth, Germany

**Keywords:** *Coenobita perlatus*, *Coenobita rugosus*, Coexistence, Competitive exclusion principle, Shell utilization, Resource partitioning

## Abstract

**Background:**

Coexistence is enabled by ecological differentiation of the co-occurring species. One possible mechanism thereby is resource partitioning, where each species utilizes a distinct subset of the most limited resource. This resource partitioning is difficult to investigate using empirical research in nature, as only few species are primarily limited by solely one resource, rather than a combination of multiple factors. One exception are the shell-dwelling hermit crabs, which are known to be limited under natural conditions and in suitable habitats primarily by the availability of gastropod shells. In the present study, we used two co-occurring terrestrial hermit crab species, *Coenobita rugosus* and *C. perlatus*, to investigate how resource partitioning is realized in nature and whether it could be a driver of coexistence.

**Results:**

Field sampling of eleven separated hermit crab populations showed that the two co-occurring hermit crab species inhabit the same beach habitat but utilize a distinct subset of the shell resource. Preference experiments and principal component analysis of the shell morphometric data thereby revealed that the observed utilization patterns arise out of different intrinsic preferences towards two distinct shell shapes. While *C. rugosus* displayed a preference towards a short and globose shell morphology, *C. perlatus* showed preferences towards an elongated shell morphology with narrow aperture.

**Conclusion:**

The two terrestrial hermit crab species occur in the same habitat but have evolved different preferences towards distinct subsets of the limiting shell resource. Resource partitioning might therefore be the main driver of their ecological differentiation, which ultimately allowed these co-occurring species to coexist in their environment. As the preferred shell morphology of *C. rugosus* maximizes reproductive output at the expense of protection, while the preferred shell morphology of *C. perlatus* maximizes protection against predation at the expense of reproductive output, shell resource partitioning might reflect different strategies to respond to the same set of selective pressures occurring in beach habitats. This work offers empirical support for the competitive exclusion principle-hypothesis and demonstrates that hermit crabs are an ideal model organism to investigate resource partitioning in natural populations.

## Background

Throughout all ecosystems, species can be found that are closely related to each other, occupy the same trophic level within the food web and share the same habitat, thus fulfilling similar ecological roles for the ecosystem [1]. When two or more species overlap to a certain degree in their biology and share a common and essential resource that is limited in supply, these species experience competition [2, 3]. This interspecific competition can occur in two forms, either via direct interference competition (i.e. fighting over resources) or via indirect exploitative competition (i.e. consumption of resources by one species makes it unavailable for second species). In ecological research, evidence for competition between two species can be provided by comparing which resources are used and which are intrinsically preferred [4].

When investigating resource utilization between co-occurring species, studies have shown that some animals that presumably compete over the same resource, actually partition the resource [5, 6]. According to the competitive exclusion principle, this resource partitioning, as a form of ecological differentiation between species, can thereby be the mechanism that allows co-occurring species to coexist in the same environment [7]. This coexistence can only be realized when each species uses a discrete subset of the limiting resource, which differs qualitatively from those of the co-occurring species [8, 9]. This premise for resource partitioning is described in the concept of limiting similarity, which states that there needs to be a limit to how similar two species can be to each other in order to stably coexist, rather than compete [5].

Such theoretical hypotheses are difficult to test using empirical research, as most animals in nature are not limited by only a single resource, but rather by a multitude of abiotic and biotic factors [10]. There exist, however, some co-occurring species, where enough evidence has been collected to suggest that they are indeed primarily limited by only one resource. Shell-dwelling hermit crabs are limited under natural conditions and in suitable habitats only by the availability of the shell resource, while food and habitat are not considered as a limiting factor [10–13]. Therefore, they appear to be suitable model organisms to investigate competition theory in empirical research.

Hermit crabs (Superfamily: Paguroidea) are characterized by an uncalcified and reduced abdomen, which they protect by utilizing mainly gastropod shells [14, 15]. As a well-fitting shell optimizes growth and maximizes clutch size [16], offers protection against predators and mechanical disruption [17, 18], and decreases the risk of desiccation in the intertidal and terrestrial species [19], hermit crabs are under constant pressure to find a well-fitting shell. The availability of empty and well-fitting shells thereby depends on the gastropod population and their mortality and hence is the limiting resource of hermit crab populations [10, 14, 20].

Co-occurring species of hermit crabs experience direct interference competition by fighting over shells in a highly ritualized behaviour and indirect exploitative competition, as the utilization of an empty shell makes it unavailable for other individuals [11, 13, 14, 21–23]. This competition can force hermit crabs to utilize shells outside their optimal fit range, resulting in a reduced fitness [10, 20, 24]. A number of studies, however, were able to demonstrate, that, contrary to the proposed shell competition, at least some co-occurring hermit crab species partition the shell resource [10, 25–27]. In these studies, the utilized gastropod shells and their morphometric parameters (e.g. size, weight) of co-occurring hermit crab species in the field were investigated and compared. It was thereby shown that co-occurring hermit crabs utilize indeed shells of different gastropod species or with different shell parameters [8, 25], although other studies suggested that the observed differences in shell utilization arise not out of different preferences [11, 21]. Therefore, it is discussed whether shell resource partitioning is indeed the mechanism of coexistence in co-occurring hermit crab species [10, 23].

One major limitation of many research approaches that investigate shell resource partitioning in hermit crabs is that the proposed preferences are based on the species identities of the gastropod shells [e.g. 20, 26]. The utilization of different shell species depends on the gastropod communities in the particular habitat and gastropod species vary between different regions [19, 24, 28, 29]. Proposing that co-occurring hermit crab species partition the shell resource by preferring different shell species is an uninformative and not universally applicable approach, because the available set of utilizable gastropod species varies between regions and does not reflect the actual preference of a hermit crab species, i.e. the same hermit crab species can prefer two completely different shell species in two different populations but in both cases select for the same morphological shell parameters.

A better approach is the comparison of preferences for different shell parameters. Determining the shell partitioning mechanism based on single shell parameters, however, is restricted, as the various shell variables are all highly intercorrelated, making it impossible to characterize a single parameter on which preferences could be based upon [30]. Using morphometric data, it was demonstrated that co-occurring hermit crab species have distinct preferences towards e.g. large shells or narrow apertures [25].

To deepen our understanding of resource partitioning as a possible driver of coexistence using empirical research on hermit crabs, it would be essential to incorporate (I) a large-scale sampling effort to pool data of multiple distinct hermit crab and gastropod populations, (II) a comparison between shell utilization patterns in the natural habitat and the intrinsic preferences towards distinct subsets of the resource and (III) a statistical analysis of the overall morphology of the different subsets of the resources, rather than a single parameter-approach.

The present study complies with the three abovementioned criteria by conducting an atoll-wide sampling that covered eleven distinct hermit crab and gastropod populations and by comparing the field data with laboratory shell preference experiments. A principal component analysis (PCA) of the shell morphometrics was then applied to compare the decisive criteria of the shell morphology between the co-occurring species. As research organisms to test competition theory, the only terrestrial hermit crab genus, *Coenobita*, was chosen, because it has already been established that the two co-occurring hermit crab species in the investigated system, *C. rugosus* and *C. perlatus*, are both primarily beach associated and unspecialized detritus feeders with no clear food preferences [31–33]. They are therefore an ideal system to test for the effect of the shell resource on coexistence, because other potentially limiting factors can be excluded upfront. The overall shell utilization in land hermit crabs has received only limited research focus in comparison to their well-studied marine counterparts [34, 35]. As terrestrial hermit crabs are restricted to one island, they inhabit and obtain the shell resource only from the surrounding coastal water [19]. Therefore, sampling multiple islands covers distinct hermit crab and gastropod populations and decreases the effect of predominant species in one island ecosystem.

## Results

### Field data

Of the 876 collected hermit crabs, 700 were identified as *C. rugosus* and 176 as *C. perlatus.* The proportion of *C. rugosus* and *C. perlatus* varied significantly between the eleven investigated islands (F = 6.2536, *df* = 10, p < 0.001). On nine out of the eleven investigated islands within the Atoll, the mean proportion of *C. rugosus* was 86.47 ± 11.64%. On one island however, only 37.05% of the collected crabs were identified as *C. rugosus*, while 62.95% were *C. perlatus*. On another island, *C. perlatus* was completely absent from the investigated plots. The proportion of *C. rugosus* (80.28 ± 7.10%) and *C. perlatus* (19.72 ± 7.10%) was not significantly different between the four investigated beach habitat types (F = 1.9196, *df* = 3, p = 0.147). The collected *C. rugosus* and *C. perlatus* had a carapace length of 6.50 ± 2.23 mm and 6.46 ± 2.71 mm, respectively. The mean carapace length of the two species did not differ statistically (Wilcoxon W = 56,344, p = 0.291). The collected *C. rugosus* inhabited gastropod shells of 90 different species (in 21 different families), while the collected *C. perlatus* inhabited gastropod shells of 41 different species (in 14 different families; see Additional file 1: Table S1). The shell species diversity index, i.e. the diversity of shell species inhabited by the two investigated hermit crab species, of *C. rugosus* was H = 3.644 and of *C. perlatus* H = 3.039. The niche width in respect to utilizable shell species was therefore B = 23.870 for *C. rugosus* and B = 12.869 for *C. perlatus* (Table 1).

**Table 1:** Comparison of the shell utilization and preferences of the two co-occurring hermit crab species

	*Coenobita rugosus*	*Coenobita perlatus*
Utilized gastropod shells	90 species (21 families)	41 species (14 families)
Cerithiid shells utilized	13.90%	32.06% (***)
Cerithiid shells selected	54.67%	56.00%
Nassariid shells utilized	28.78%	18.49%
Nassariid shells selected	64.00%	65.33%
Naticid shells utilized	14.09%	4.22% (***)
Naticid shells selected	56.00%	20.00% (***)
Strombid shells utilized	12.77%	39.52% (***)
Strombid shells selected	25.33%	58.67% (***)
Shell diversity Shannon H	3.644	3.039
Niche width B in respect to shell species	23.870	12.869

Asterisks (***p < 0.001) indicate significant differences in the proportional utilization or selection of the respective shell type between the two hermit crab species, *C. rugosus* and *C. perlatus*

The proportional utilization of the investigated shell types differed significantly between *C. rugosus* and *C. perlatus* (Table 1). Proportionally more *C. rugosus* inhabited naticid shells than *C. perlatus* (p = 0.003), while proportionally more *C.* *perlatus* inhabited cerithiid (p < 0.001) and strombid shells (p < 0.001). No differences were found in the number of inhabited nassariid shells between *C. rugosus* and *C. perlatus* (p = 0.237; Table 1).

### Shell preference experiments

The mean carapace length of the 150 tested *C. rugosus* was 6.25 ± 1.43 mm and of the 150 tested *C. perlatus* 6.42 ± 1.42 mm (mean ± standard deviation). The size of the tested hermit crab in the laboratory experiment did not differ statistically between the two species (Wilcoxon W = 12,207, p = 0.199).

The two terrestrial hermit crabs *C. rugosus* and *C. perlatus* had significantly different shell preferences for the tested gastropod shells (Table 1, Additional file 2: Table S2). *C. perlatus* selected strombid shells significantly more often than *C. rugosus* (p < 0.001) and *C. rugosus* selected naticid significantly more often than *C. perlatus* (p < 0.001). No differences existed for the number of selected cerithiid (p = 1.000) and nassariid shells (p = 1.000) between the two hermit crab species.

### Morphometric analysis of gastropod shells

The five investigated morphometric parameters (shell length, shell width, aperture length, aperture width, shell weight) of the utilized gastropod shells differed significantly between the four investigated gastropod shell types (F = 71.505, *df *= 3, p < 0.001) and between the two hermit crab species (F = 16.080, *df* = 1, p < 0.001).

**Fig. 1:**
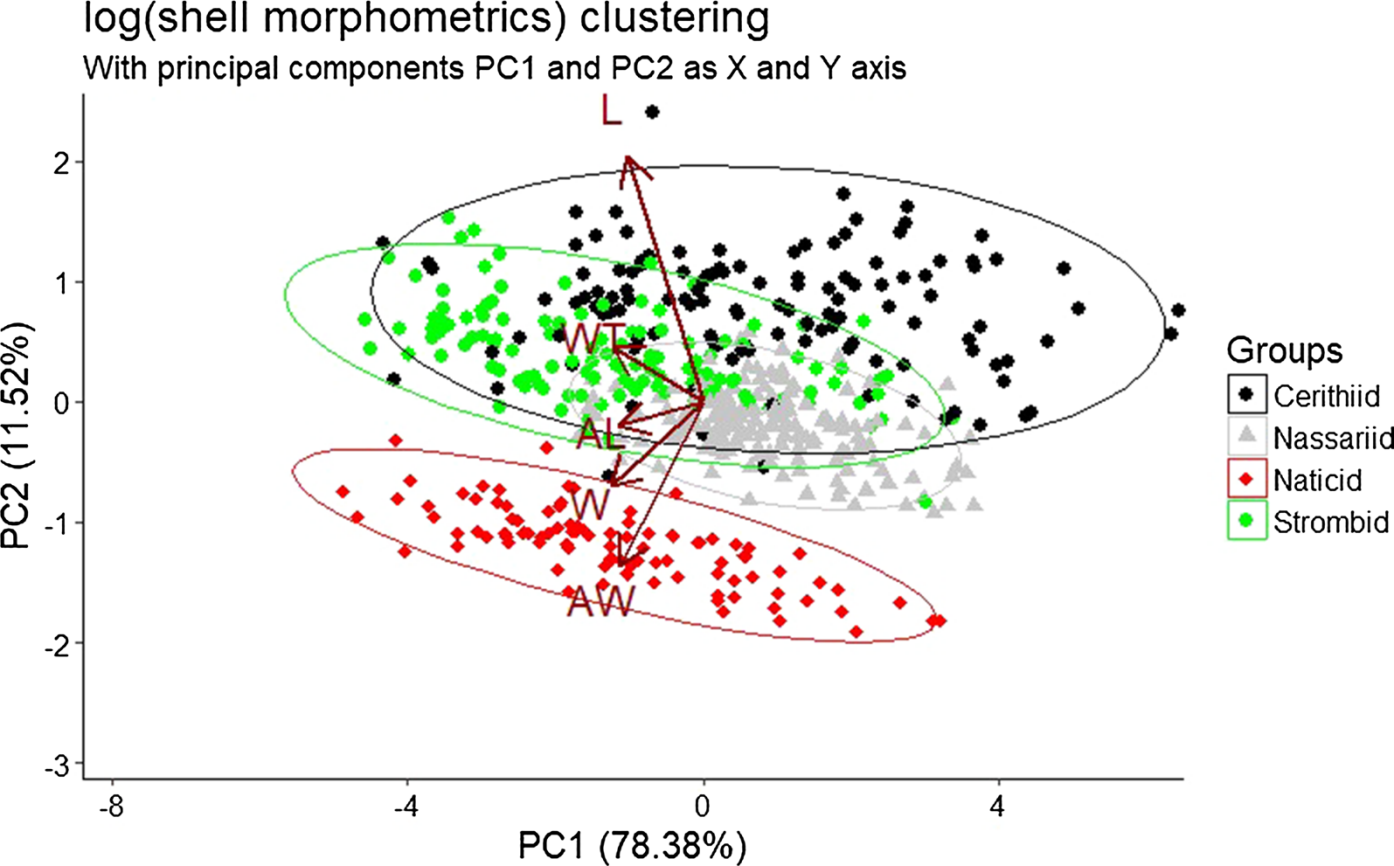
The shell morphology of the four most utilized gastropod shell types. The principal component analysis is based on the five log-transformed morphometric parameters (*AL* aperture length, *AW* aperture width, *L* length, *W* width, *WT* weight). Each data point represents a single shell, colours resemble the different shell types

The first three principal components of the PCA, comparing the morphometric parameters, explained 96.47% of the total variance and were therefore used for further analysis (Fig. 1). Principal component 1 (PC1) correlates with all five morphometric parameters, suggesting that all five parameters vary together. PC2 is primarily a measure for shell length (correlation 0.784) and aperture width (correlation − 0.526) and can be viewed as an overall descriptor of the shell shape with high values of PC1 indicating an elongated and narrow shell shape, while low values of PC2 indicate a short and bulbous shell shape. PC3 negatively correlates with aperture length (correlation − 0.851) and can be viewed as a measure of how elongated the shell aperture is Table 2.

**Table 2:** Comparison of the shell morphology of the four most utilized gastropod shell types and the two hermit crab species

	PC 1	PC 2	PC 3
Shell length	− 0.396	0.784	0.080
Shell width	− 0.485	− 0.265	0.016
Aperture length	− 0.438	− 0.078	− 0.851
Aperture width	− 0.437	− 0.526	0.362
Shell weight	− 0.472	0.174	0.370
Shell thickness	− 0.329	− 0.804	0.046
Cerithiid shells	0.874 (A)	0.765 (A)	0.372 (A)
Nassariid shells	0.839 (A)	− 0.200 (B)	0.268 (A)
Naticid shells	− 1.198 (B)	− 1.189 (C)	0.056 (B)
Strombid shells	− 1.195 (B)	0.384 (D)	− 0.791 (C)
*Coenobita rugosus*	0.151 (A)	− 0.134 (A)	0.046 (A)
*Coenobita perlatus*	− 0.479 (B)	0.424 (B)	− 0.146 (B)

Principal components (PC) of the PCA are based on five morphometric parameters of the four utilized gastropod shell types. Significant differences between the mean PC values for each shell type are indicated by different letters behind the PC value, same letters indicate no statistical difference between the PC values of the respective shell types

The four gastropod shell types differed significantly in PC1 (F = 60.96, *df *= 3, p < 0.001), PC2 (F = 548.1, *df *= 3, p < 0.001) and PC3 (F = 307.8, *df *= 3, p < 0.001). Tukey HSD post hoc test indicated significant differences in PC1 between all pairwise comparisons (p < 0.001), apart from nassariid-cerithiid (p = 0.997) and strombid-naticid shells (p = 0.999). PC2 was significantly different in all pairwise comparisons (p < 0.001 in all comparisons). PC3 was significantly different in all comparisons (p < 0.001), apart from one non-significant difference in the pairwise comparison of nassariid and cerithiid shells (p = 0.051; Table 2).

All three principal components of the shell parameters differed significantly between the two hermit crab species (PC1: F = 9.819.3, *df *= 1, p = 0.001; PC2: F = 57.01, *df *= 1, p < 0.001; PC3: F = 92.14 *df *= 1, p < 0.001; Additional file 3: Fig. S1).

## Discussion

According to the competitive exclusion principle, ecological differentiation is the premise for coexistence in co-occurring species [7]. This ecological differentiation can be realized by partitioning the limiting resource between two species [9]. In the present study, the utilization of the limiting resource of two co-occurring hermit crab species was investigated to study the relevance of resource partitioning as a driver of coexistence. In natural populations, the two co-occurring hermit crabs *C. rugosus* and *C. perlatus* utilized different gastropod shell species. These differences in the shell utilization of the two hermit crab species arise out of different preferences towards different shell types. Together with the morphometric analysis, the presented data suggest that the two hermit crab species are not in competition over the limited shell resource but have evolved different preferences towards distinct subsets of the shell resource, which ultimately could enable both species to coexist in their habitat.

Coexistence of co-occurring marine hermit crabs has been suggested to arise out of a combination of resource and habitat partitioning [10, 14]. Terrestrial hermit crabs are more restricted in their habitat choice, as especially small islands offer only little heterogeneity in the beach environment [36–39]. Although *C. perlatus* was overall less abundant than *C. rugosus*, there relative proportions did not differ between the four present beach habitat types. As both species are known to be primarily beach-associated and rarely occurring in the densely vegetated inland [40–44], the high overlap of both species in the beach habitats suggests that habitat partitioning is not a driver of coexistence in these two species.

Partitioning of or competition over the food resource can also be excluded as a driver for coexistence, as previous studies demonstrated that *C. rugosus* and *C. perlatus* are both unspecific detritus feeders with no clear food preference [32, 43] and not limited by food availability [10, 14, 22].

As habitat and food resource partitioning appears to play a minor role for *C. rugosus* and *C. perlatus*, the possible mechanism for coexistence might arise out of shell resource partitioning. The morphometric analysis of the utilized shells in the field suggests that *C. rugosus* utilizes shells with a small and globose morphology, while *C. perlatus* utilizes shells with a large, elongated and narrow morphology. These utilization patterns arise indeed out of different intrinsic preferences towards the respective shell morphology, as *C.* *rugosus* selected for the short and globose naticid shells, while C*. perlatus* selected for the large and elongated strombid shells in the laboratory experiments. The determined preferences towards a certain shell morphology lay in concordance with previous studies, which reported *C. rugosus* to utilize mainly Muricidae, Neritidae or Turbinidae shells, which also have a globose morphology, and *C. perlatus* to utilize mainly the elongated cerithiid shells [35, 40, 43–45]. This overall similarity further underlines that not the shell species itself is the decisive criteria in the shell selection process, but rather the overall morphology of the present shell, described by the principal components of the morphometric data. The utilized shells found in the natural populations were overall fairly eroded and showed no striking variations in colour or ornamentation but appeared rather uniform pale and smooth, independent of the gastropod species. Therefore, preferences towards certain shell colours or ornamental features like spines can be excluded as further decisive factors in shell selection of the investigated hermit crab species. As gastropod communities vary between different regions, the adaptive mechanism in shell selection behaviour is therefore not the evolution of preferences towards species (although at least one hermit crab species is known utilizing only one shell species, *Calcinus seurati* [14, 20]), but rather of preferences towards certain shell morphologies [46].

The two investigated hermit crab species apparently have evolved different shell preferences towards distinct subsets of the shell resource. These intrinsic preferences could hint towards differing strategies of the two hermit crab species to respond to the same overall selective pressures [47, 48]. Heavy and elongated shells with a narrow aperture, like the strombid shells, offer optimal protection against desiccation and predation, but limit clutch size and increase energy expenditure during locomotion due to a reduced internal volume and increased weight [8, 16, 20, 25]. Light-weight and voluminous shells, like the naticid shells, allow a greater dispersal and are advantageous for burrowing, but cannot retain water efficiently and offer less protection against predation [27, 40, 49]. As different shell preferences might represent different strategies to respond to selective pressures from the same environment, *C. perlatus* might has evolved a strategy to reduce desiccation- and predation-related mortality at the expense of an increased energy expenditure and limited clutch size [48]. *C. rugosus* has evolved a strategy to maximize reproductive output at the expense of an increased susceptibility for desiccation and predation.

Further research is needed to test, whether the observed shell resource partitioning in the two co-occurring hermit crab species is the cause or the effect of the proposed ecological differentiation in respect to their life-history strategy and if the utilization of different subsets of the shell resource can even be a driver of speciation in hermit crabs.

In either way, it is shown that the utilization of distinct subsets of the limiting resource can drive ecological differentiation, which then ultimately enables two species to coexist [7, 9]. It is thereby demonstrated that co-occurring hermit crabs are indeed suitable model organisms to empirically investigate competition and coexistence theory, as their limitation by primarily one resource offers controllable and empirically testable conditions for investigating natural and intrinsic behaviour of resource partitioning.

## Conclusion

Overall, our research investigated the mechanism of resource partitioning as a driver of coexistence and demonstrated that two co-occurring species of terrestrial hermit crabs have evolved intrinsic preferences towards distinct subsets of the shell resource, which attenuates interspecific competition over the limiting resource in natural populations. As the preferred shell morphologies of the two hermit crab species either maximize reproductive output or minimize predation risk, the two hermit crab species might have evolved different strategies to respond to the overall selective pressures in their natural habitat.

These findings offer empirical support for theoretical hypotheses on competition theory and mechanisms of coexistence in ecology. By discussing different life-history strategies, associated with the observed resource partitioning, the presented model system using hermit crabs can form the basis for future research on mechanisms of coexistence and speciation.

## Methods

### Field data

Hermit crabs were collected on the beaches of eleven coral islands, distributed over the Lhaviyani (Faadhippolhu) Atoll, Republic of Maldives. Sampling was carried out between 03/02/2017 and 10/03/2017, always in the time from 2 h before low tide until absolute low tide. On each island, hermit crabs were collected in six plots with 10 m length (measured along the current drift line) and 2 m width (measured perpendicular to the current drift line). The habitat structure of each plot was assigned in four different beach habitat types: (1) fine sand beach, (2) fine sand beach interspersed with small coral and rock fragments, (3) fine sand beach interspersed with larger boulders and (4) predominantly rock-covered beach. The collected hermit crabs were transferred to the laboratory and removed from their shell by carefully heating the apex of the shell above an open flame. This is a standard procedure when investigating hermit crabs and leaves the animal without injuries [27, 49]. Afterwards, the hermit crab and their corresponding shell were photographed on millimetre paper (Nikon D5000 mounted with Nikon AF-S Nikkor 18–105 mm, 1:3.5–5.6, Nikon Corp., Tokyo, Japan.) and identified using identification keys [50–54]. The weight of the shell was measured using a fine scale (TS-300 300 g × 0.01 g, G&G GmbH, Neuss, Germany).

The carapace length of the hermit crabs and the morphometric parameters of their corresponding shell were determined using ImageJ 1.49b (Rasband, W.S., ImageJ, U. S. National Institutes of Health, Bethesda, Maryland, USA, http://imagej.nih.gov/ij/, 1997–2015). Shell length was measured from the shell’s apex to the siphonal notch—if present—or otherwise to the lower end of the aperture. Shell width was measured perpendicular to the longitudinal axis of the shell at the broadest section. Shell aperture length was measured from the anterior to the posterior canal of the aperture and aperture width was measured perpendicular to the aperture length between the outer lip and the columellar fold at the broadest section.

**Fig. 2:**
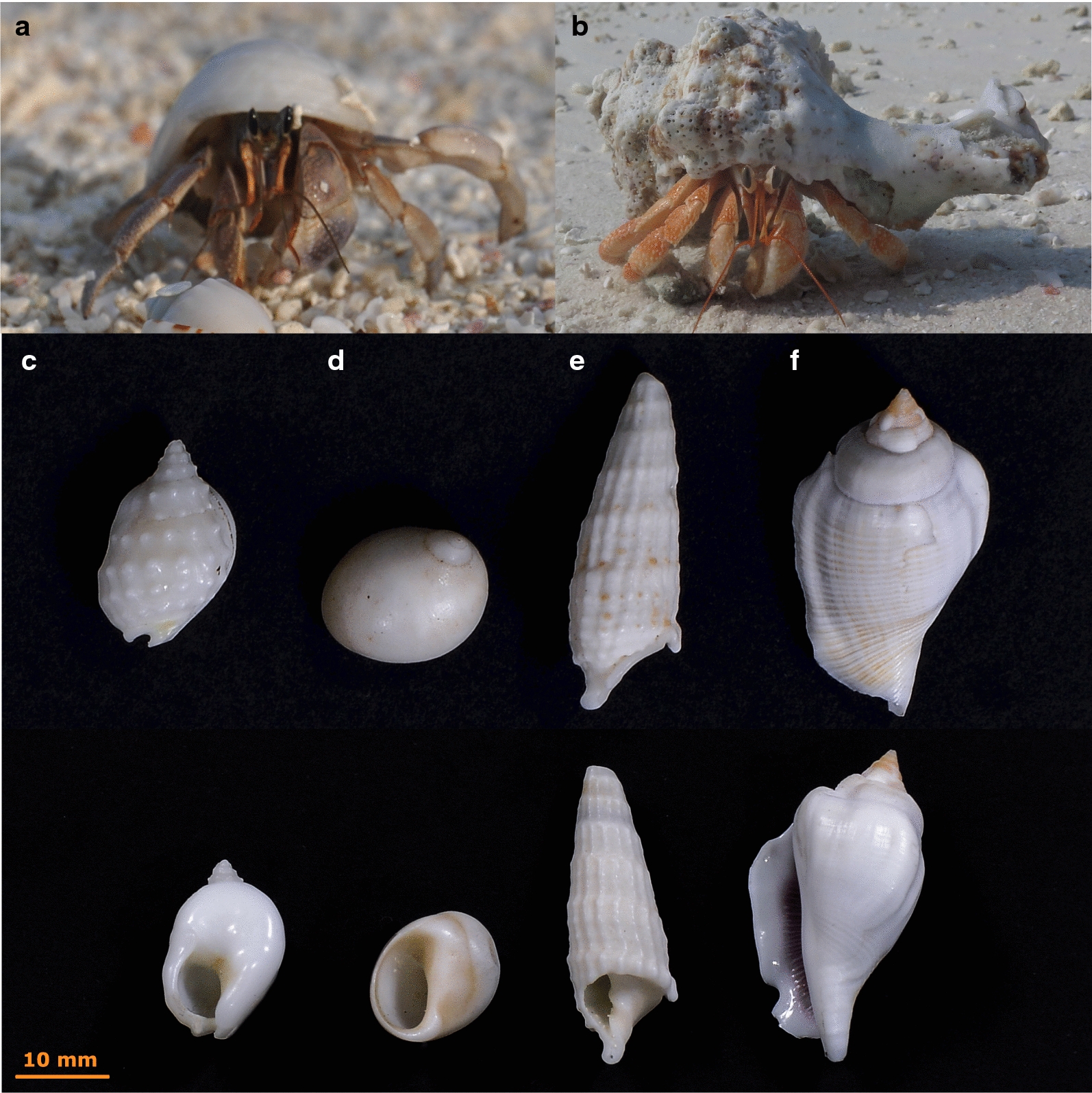
The two co-occurring hermit crab species and the four most commonly utilized gastropod shell types. On the top, the two tested hermit crab species, *Coenobita rugosus* (**a**) and *C. perlatus* (**b**) and below the four different shell types utilized, i.e. nassariid (**c**; here depicted: *Nassarius* *variciferus*), naticid (**d**; here depicted *Polinices* *mammilla*), cerithiid (**e**; here depicted *Rhinoclavis aspera*) and strombid shells (**f**; here depicted *Gibberulus gibberulus*)

Statistical analysis was performed using R 3.5.1. [55] Differences in the number of shells utilized for a given shell species between *C. rugosus* and *C. perlatus* were tested for the four most abundant gastropod families in the plots, i.e. strombid shells (246 specimen), nassariid shells (196 specimen), cerithiid shells (166 specimen) and naticid shells (141 specimen; Fig. 2). Statistical comparison in the number of utilized shells of each of the four shell types between the two collected hermit crab species were analysed using Fisher’s exact test [56]. Levels of significance were adjusted using Bonferroni–Holm-correction. The relative abundance of the two hermit crab species was calculated and statistically compared between the four investigated beach habitat type and between the eleven investigated coral islands using non-parametric multivariate analysis (PERMANOVA) with 999 permutations, implemented in the vegan package of R [57]. The diversity of shell species occupied by the two hermit crab species was calculated using the Shannon-Index H. Based on the number of inhabited shells from the two hermit crab species, the niche breadth (B) with respect to shell species inhabited was calculated using

$$B = \frac{1}{{\sum {(p_{i}^{2} )} }}$$

where p_i_ is the proportion of crabs (*C. rugosus* or *C. perlatus*) found in shells of the gastropod species I [13]. The sizes of the two sampled hermit crab species were statistically compared using Wilcoxon test.

### Shell preference experiments

150 hermit crabs of each of the two species *C. rugosus* and *C. perlatus* and 150 cerithiid, nassariid, naticid and strombid shells were collected on the beaches of Naifaru, Lhaviyani (Faadhippolhu) Atoll, Republic of Maldives from 16/03 to 20/03/2017. The collected hermit crabs were transferred into the laboratory and removed from their shell. After removing the crab out of its shell, the carapace length was measured using a ruler and the size of the crab with its corresponding shell was noted.

One hermit crab (without its shell) of a given size was then transferred into a 45-cm diameter test arena, filled 2 cm with sand from the adjacent beaches, and left to acclimatise for 5 min. After acclimatisation, two of the four tested shell types, were placed next to each other on a random place inside the test arena with the aperture facing upwards. For each tested hermit crab of a given size, two empty gastropod shells were presented that were formerly inhabited by a hermit crab with the same size of the one tested in the arena (e.g. a 1 cm-sized hermit crab was offered two shells that were formerly inhabited by 1 cm-sized crabs). This procedure was conducted to ensure that both presented shells were principally utilizable for the tested hermit crab of a given size. For *C. rugosus* and *C. perlatus* each combination of two shell species (strombid vs. naticid, strombid vs. nassariid, strombid vs. cerithiid, naticid vs. nassariid, naticid vs. cerithiid, nassariid vs. cerithiid) was tested 25 times (n = 25). One hour after presenting the two empty gastropod shells, the utilized shell type was noted and the hermit crab together with both shells transferred back to its original habitat. If no shell had been utilized by the tested hermit crab after 1 h, the experiment was terminated and the crab, as well as both shells, excluded from the experiment and transferred back to the original habitat.

The carapace lengths between the two tested hermit crab species was statistically compared using the Wilcoxon test. Preferences for the investigated shell species, between the two hermit crab species were analysed using Fisher’s exact test. Levels of significance were adjusted using Bonferroni–Holm-correction.

### Morphometric analysis of gastropod shells

Differences in the five morphometric parameters between the four different gastropod types and the two hermit crab species were compared using non-parametric multivariate analysis (PERMANOVA) with 999 permutations. One principal component analysis (PCA) was performed with log-transformed values of the five morphometric parameters. Statistical differences between the principal components of the four shell types and the two hermit crab species were analysed using ANOVA and Tukey HSD post hoc tests.

## Supplementary information

**Additional file 1: Table S1.** Gastropod species utilized by the two co-occurring hermit crab species, *C. rugosus* and *C. perlatus*, in natural populations (*N* = 11).

**Additional file 2: Table S2.** Outcome of the two-choice preference experiments. Each combination of shells was tested 25 times (*N* = 25).

**Additional file 3: Fig. S1.** Shell partitioning of the two hermit crab species. PCA calculation based on the five investigated morphometric parameters of their utilized gastropod shells. (AL: aperture length, AW: aperture width, L: length, W: width, WT: weight). Each data point represents a single shell, colours resemble the two co-occurring hermit crab species (black: *C. perlatus*, grey: *C. rugosus*).

## Data Availability

The datasets generated during this study are available from the corresponding author on reasonable request.
