# Supplementary material for: Shell resource partitioning as a mechanism of coexistence in two co-occurring terrestrial hermit crab species
Source: BMC Ecol. 2020 Jan 16;20:1. doi: 10.1186/s12898-019-0268-2 (PMC6964008; doi:10.1186/s12898-019-0268-2)
Supplement: Supplementary file 1 — Additional file 1: Table S1. Gastropod species utilized by the two co-occurring hermit crab species, C. rugosus and C. perlatus, in natural populations (N = 11). [file 12898_2019_268_MOESM1_ESM.docx]

**Table S1:** Gastropod species utilized by the two co-occurring hermit crab species, *C. rugosus* and *C. perlatus*, in natural populations (*N* = 11).

| **Gastropod species** | ***Coenobita rugosus*** | ***Coenobita perlatus*** |
| --- | --- | --- |
| *Angaria rugosa* | 1 | 0 |
| *Bursa bufonia* | 4 | 0 |
| *Canarium labiatum* | 4 | 0 |
| *Canarium mutabile* | 22 | 13 |
| *Casmaria erinaceus* | 4 | 0 |
| *Casmaria ponderosa* | 5 | 0 |
| *Cerithium columna* | 27 | 19 |
| *Cerithium echinatum* | 1 | 2 |
| *Cerithium nesioticum* | 1 | 0 |
| *Cerithium nodulosum* | 13 | 0 |
| *Cerithium punctatum* | 1 | 0 |
| *Cerithium rostratum* | 5 | 2 |
| *Chicoreus* cf. *axicornis* | 2 | 0 |
| *Chicoreus* cf. *brunneus* | 1 | 1 |
| *Clypeomorus* cf. *irrorata* | 2 | 0 |
| *Columbella sp.* | 1 | 0 |
| *Coralliophila erosa* | 24 | 1 |
| *Coralliophila violacea* | 1 | 0 |
| *Cymatium gemmatum* | 1 | 0 |
| *Cymatium hepaticum* | 0 | 2 |
| *Cymatium labiosum* | 5 | 0 |
| *Cymatium mundum* | 6 | 1 |
| *Cymatium nicobarium* | 15 | 7 |
| *Drupa ricinus* | 3 | 0 |
| *Drupa rubusidaeus* | 2 | 2 |
| *Drupella* cf. *margariticola* | 29 | 6 |
| *Drupella cornus* | 13 | 4 |
| *Drupella fragum* | 1 | 0 |
| *Drupina lobata* | 4 | 2 |
| *Euplica turturina* | 1 | 0 |
| *Gibberulus gibberulus* | 51 | 36 |
| *Gutturnium muricinum* | 4 | 1 |
| *Gyrineum bituberculare* | 1 | 0 |
| *Gyrineum gyrinum* | 1 | 0 |
| *Gyrineum natator* | 1 | 0 |
| *Harpa amouretta* | 1 | 1 |
| *Hemipolygona* cf. *bonnieae* | 3 | 0 |
| *Latirus polygonus* | 3 | 0 |
| *Littoraria undulata* | 4 | 1 |
| *Malea pomum* | 1 | 1 |
| *Mammilla melanostoma* | 6 | 0 |
| *Mammilla simiae* | 1 | 0 |
| *Mancinella alouina* | 7 | 0 |
| *Mancinella echinata* | 18 | 1 |
| *Mancinella echinulata* | 1 | 0 |
| *Modulus tectum* | 24 | 2 |
| *Monoplex aquatile* | 19 | 5 |
| *Monoplex* cf. *parthenopeus* | 3 | 1 |
| *Morula aspera* | 1 | 0 |
| *Myurella affinis* | 10 | 3 |
| *Nassa francolina* | 2 | 0 |
| *Nassarius* cf. *reeveanus* | 4 | 0 |
| *Nassarius distortus* | 13 | 2 |
| *Nassarius echinatus* | 24 | 6 |
| *Nassarius granifer* | 73 | 11 |
| *Nassarius horridus* | 28 | 6 |
| *Nassarius papillosus* | 7 | 0 |
| *Nassarius sp.* | 0 | 1 |
| *Nassarius variciferus* | 3 | 1 |
| *Natica vitellus* | 0 | 1 |
| *Neothais marginatra* | 1 | 0 |
| *Nerita albicilla* | 1 | 0 |
| *Nerita costata* | 7 | 1 |
| *Nerita plicata* | 0 | 1 |
| *Nerita polita* | 9 | 0 |
| *Notocochlis gualtieriana* | 1 | 0 |
| *Oxymeris felina* | 1 | 0 |
| *Oxymeris maculata* | 2 | 1 |
| *Peristernia nassatula* | 5 | 0 |
| *Peristernia ustulata* | 1 | 1 |
| *Pleuroploca* cf. *trapezium* | 2 | 0 |
| *Polinices mammilla* | 74 | 6 |
| *Polinices melanostomoides* | 1 | 0 |
| *Pollia rawsoni* | 1 | 0 |
| *Psilaxis radiatus* | 1 | 0 |
| *Rhinoclavis articulata* | 7 | 1 |
| *Rhinoclavis aspera* | 9 | 5 |
| *Rhinoclavis kochi* | 2 | 0 |
| *Rhinoclavis sinensis* | 13 | 12 |
| *Semiricinula tissoti* | 2 | 0 |
| *Tectonatica violacea* | 1 | 0 |
| *Thais sp.* | 0 | 1 |
| *Thalessa* cf. *aculeata* | 1 | 0 |
| *Thalessa virgata* | 2 | 0 |
| *Tonna allium* | 2 | 0 |
| *Tonna perdix* | 1 | 1 |
| *Trochus* cf. *kochii* | 1 | 0 |
| *Turbo argyrostomus* | 10 | 0 |
| *Turbo petholatus* | 1 | 0 |
| *Turridrupa cincta* | 1 | 1 |
| *Turris crispa* | 2 | 0 |
| *Turritriton labiosus* | 2 | 0 |
| *Vanikoro cancellata* | 1 | 0 |
| *Vexilla vexillum* | 1 | 0 |
| *Vitularia sp.* | 1 | 0 |
