# Supplementary material for: Shell resource partitioning as a mechanism of coexistence in two co-occurring terrestrial hermit crab species
Source: BMC Ecol. 2020 Jan 16;20:1. doi: 10.1186/s12898-019-0268-2 (PMC6964008; doi:10.1186/s12898-019-0268-2)
Supplement: Supplementary file 2 — Additional file 2: Table S2. Outcome of the two-choice preference experiments. Each combination of shells was tested 25 times (N = 25). [file 12898_2019_268_MOESM2_ESM.docx]

**Table S2: Outcome of the two-choice preference experiments.** Each combination of shells was tested 25 times (*N* = 25).

| **Pairwise comparison** | ***Coenobita rugosus*** | ***Coenobita perlatus*** |
| --- | --- | --- |
| nassariid – naticid | 16 – 9 | 23 – 2 |
| nassariid – strombid | 18 – 7 | 13 – 12 |
| nassariid - cerithiid | 14 – 11 | 13 – 12 |
| naticid – strombid | 18 – 7 | 7 – 18 |
| naticid – cerithiid | 15 – 10 | 6 – 19 |
| cerithiid – strombid | 20 – 5 | 11 – 14 |
