# Supplementary material for: Shell resource partitioning as a mechanism of coexistence in two co-occurring terrestrial hermit crab species
Source: BMC Ecol. 2020 Jan 16;20:1. doi: 10.1186/s12898-019-0268-2 (PMC6964008; doi:10.1186/s12898-019-0268-2)
Supplement: Supplementary file 3 — Additional file 3: Fig. S1. Shell partitioning of the two hermit crab species. PCA calculation based on the five investigated morphometric parameters of their utilized gastropod shells. (AL: aperture length, AW: aperture width, L: length, W: width, WT: weight). Each data point represents a single shell, colours resemble the two co-occurring hermit crab species (black: C. perlatus, grey: C. rugosus). [file 12898_2019_268_MOESM3_ESM.docx]

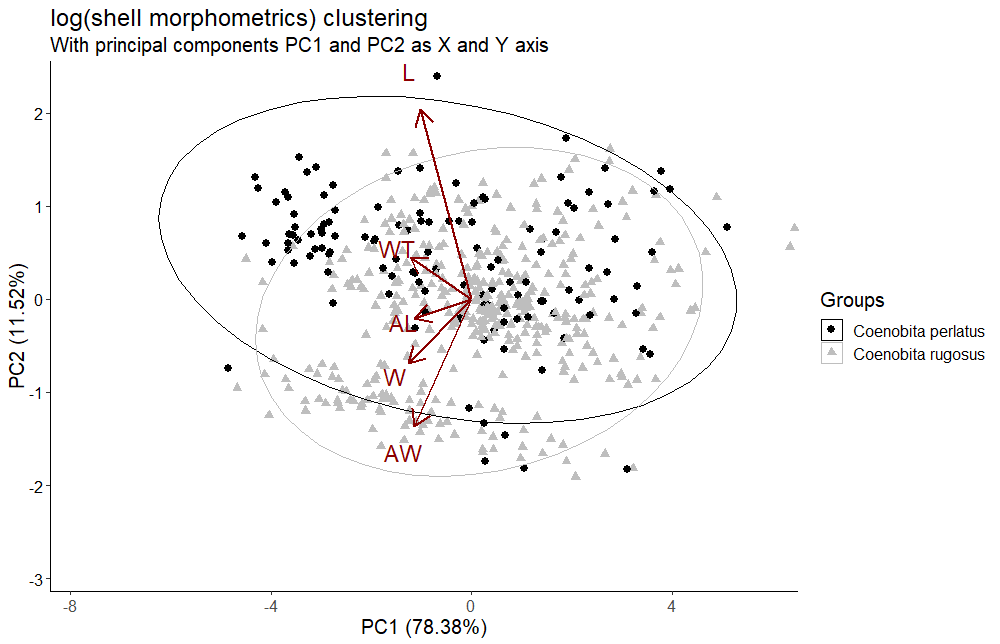


**Figure S1: Shell partitioning of the two hermit crab species**. PCA calculation based on the five investigated morphometric parameters of their utilized gastropod shells. (AL: aperture length, AW: aperture width, L: length, W: width, WT: weight). Each data point represents a single shell, colours resemble the two co-occurring hermit crab species (black: *C. perlatus*, grey: *C. rugosus*).
